# Autoreactivity and broad neutralization of antibodies against HIV-1 are governed by distinct mutations: Implications for vaccine design strategies

**DOI:** 10.3389/fimmu.2022.977630

**Published:** 2022-11-18

**Authors:** Xiaojun Li, Dongmei Liao, Zhengyang Li, Jixi Li, Marilyn Diaz, Laurent Verkoczy, Feng Gao

**Affiliations:** ^1^ Department of Medicine, Duke University Medical Center, Durham, NC, United States; ^2^ Department of Immunology, Duke University Medical Center, Durham, NC, United States; ^3^ School of Life Sciences, Fudan University, Shanghai, China; ^4^ Applied Biomedical Science Institute, San Diego, CA, United States; ^5^ Institute of Molecular and Medical Virology, School of Medicine, Jinan University, Guangzhou, Guangdong, China; ^6^ Guangdong Provincial Key Laboratory of Virology, Institute of Medical Microbiology, Jinan University, Guangzhou, Guangdongg, China

**Keywords:** HIV-1, broadly neutralizing antibody, autoreactivity, poly reactivity, mutation

## Abstract

Many of the best HIV-1 broadly neutralizing antibodies (bnAbs) known have poly-/autoreactive features that disfavor normal B cell development and maturation, posing a major hurdle in developing an effective HIV-1 vaccine. Key to resolving this problem is to understand if, and to what extent, neutralization breadth-conferring mutations acquired by bnAbs contribute to their autoreactivity. Here, we back-mutated all known changes made by a prototype CD4 binding site-directed bnAb lineage, CH103-106, during its later maturation steps. Strikingly, of 29 mutations examined, only four were crucial for increased autoreactivity, with minimal or no impact on neutralization. Furthermore, three of these residues were clustered in the heavy chain complementarity-determining region 2 (HCDR2). Our results demonstrate that broad neutralization activity and autoreactivity in the CH103-106 bnAb lineage can be governed by a few, distinct mutations during maturation. This provides strong rationale for developing immunogens that favor bnAb lineages bearing “neutralization-only” mutations into current HIV-1 vaccine designs.

## Introduction

The HIV-1 genome is highly mutable, yielding upwards of 30% amino acid differences between envelope glycoproteins (Envs) from different subtypes (1). Thus, an effective HIV-1 vaccine will need to induce broadly neutralizing Abs (bnAbs) which can neutralize diverse HIV-1 variants (2, 3). The isolation of now well over 100 bnAbs has led to the identification of seven conserved targets in the HIV-1 Envelope (Env): the CD4 binding site (CD4bs), the Env trimer apex in the V1V2 region, the high-mannose patch in the V3 loop, the gp120-gp41 interface, the fusion peptide (FP) domain, the silent face center, and the membrane-proximal external region (MPER) (4–6). Studies demonstrate that passive transfer of bnAbs prevent acquisition of infection (7–10), indicating that bnAbs can be highly effective if they are induced by vaccines. However, the bnAbs identified thus far have been observed only in certain individuals after prolonged infection, generally after 2-4 years (11–14). Therefore, reproducibly achieving broad neutralization within a practical vaccine schedule in a majority of individuals is not only critical for the development of an effective vaccine but by definition, would need to represent a dramatic improvement over natural infection. To accomplish this, we must first understand what factors contribute to the fact that bnAbs are elicited in such a suboptimal and delayed fashion during natural infection.

Characterization of bnAbs has revealed that they tend to share at least two of the following unusual sets of traits: *in vitro* poly-/autoreactivity, long heavy chain third complementarity determining region 3 (HCDR3), and exceptionally high levels of somatic mutations (3, 15–23). Mounting evidence from a multitude of bnAb knock-in models clearly demonstrate such *in vitro* poly-/autoreactivities are sufficient to trigger profound tolerance controls *in vivo*, at least at one (but often more) negative B-cell selection checkpoints during bnAb lineage development (i.e., in unmutated bnAb precursor B-cells, their partially evolved mutated intermediates and/or fully evolved B cells that can secrete bnAbs) (3, 20–24). Thus, host tolerance controls, triggered by bnAb lineage poly-/autoreactivities, provide a rational explanation for why many (if not most) of the broadest and most potent bnAbs identified can be only sub-optimally elicited during prolonged HIV-1 infection (12, 14, 24–27). Similarly, somatic hypermutation (SHM) levels acquired in some bnAb lineages during natural infection can reach up to 50% amino acid changes, and most crucially, the minimal number required for breadth and potency is invariably still far higher for all bnAbs than that typically elicited by conventional vaccines (28), likely representing another factor limiting protective HIV-1 responses. Thus, a deeper understanding of the functional and temporal relationship between high SHM levels and tolerizing poly-/autoreactivities for development of broad neutralization is critical (29–36). In this study, we address two related, pressing questions towards achieving this level of understanding: 1) is poly-/autoreactivity always required during bnAb lineage development for gaining neutralization breadth? and 2) if not, to what extent do somatic mutations that govern poly-/autoreactivity overlap with those responsible for neutralization breadth?

Here, to formally address these questions, we sought to identify somatic mutations linking bnAb activity with poly-/autoreactivity during the maturation of CH103-106, a prototype clonally-related lineage of CD4bs-targeting (“germline encoded HCDR3-binder” subclass) bnAbs isolated from a chronically HIV-infected subject (13). We have previously extensively characterized this bnAb lineage and thus have in-depth knowledge of the maturation pathways leading to neutralization breadth and potency. In the present study, we interrogate the late (breadth-conferring) steps of this lineage’s pathways, in order to ascertain whether neutralization and autoreactivity can be decoupled during bnAb maturation, which in turn has important implications for vaccine design. Specifically, if poly-/autoreactivity acquired through Ig somatic mutation does not impact neutralization breadth and potency, then it is possible and crucial to decouple neutralization breadth from poly-/autoreactivity by focusing immunogen design efforts to target neutralization specificity while minimizing autoreactivity. If instead we find that poly-/autoreactivity is required for these bnAbs, then strategies to selectively overcome their survival disadvantage such as providing unusually strong BCR and specific CD4 T-cell signals may be required (37, 38).

## Materials and methods

### Site-directed mutagenesis

Three sets of mutations in closely related CH103-106 lineage Ab pairs were made to systemically dissect contributions by individual residue substitutions on poly-/autoreactivity and neutralization differences. In the first set, mutations N60S and E64K were individually introduced in the V_H_ region of the non-poly-/autoreactive intermediate IA2, to reflect the only two amino acid (aa) substitutions between it and the highly poly-/autoreactive intermediate IA1. In the second set, 17 aa substitutions (L11V, Y32T, Q39L, V42G, N60S, E64K, S65G, T68S, V69I, N76D, Y91F, F99Y, D101R, Q105R, T110S, A112T, and S113A) were individually introduced in the V_H_ region of the weakly poly-/autoreactive intermediate IA3, corresponding to differences between it and the fully poly-/autoreactive intermediate IA1. In the third set, three aa differences (T23S, E56H and S107I) were introduced in the V_H_ region of the less poly-/autoreactive, fully mature CH103 bnAb, as well as six aa differences (T31V, E45D, I48M, F49Y, I81T and D85E) and one 3-aa insertion (DKV) in the V_L_ region, to reflect the total of 10 residue differences between it and another fully mature, but more poly-/autoreactive lineage bnAb, CH106. Mutagenesis was carried out using the Quick-Change II Site-Directed Mutagenesis kit, following the manufacturer’s protocol (Agilent Technologies, Santa Clara, CA) (39). All final Ab mutants were confirmed by sequencing their entire heavy and light chains.

### Neutralization assay

Neutralization activity was measured as a reduction in luciferase activity after a single round infection of TZM-bl cells, as previously described (40–42). Neutralization potency and breadth of recombinant monoclonal Abs was determined by testing eight serial 3-fold dilutions, starting at 50 µg/ml, against 15 HIV-1 Env pseudo-viruses (13 tier-2 viruses and 2 tier-1 viruses) on TZM-bl cells (43–45). Briefly, pseudo-viruses were incubated with Abs for 1 hour at 37°C. TZM-bl cells were added and cells were maintained at 37°C with 5% CO_2_ for 48 hours. Viral infectivity was determined by measuring firefly luciferase activity with Britelite Plus reagent (Perkin-Elmer, Waltham, MA) on a luminometer. The 50% inhibitory concentration (IC_50_) was defined as the Ab concentration at which relative luminescence units (RLU) were reduced by 50% compared with RLU in virus control wells, after subtracting background RLU in cell control wells (13).

### Expression and purification of recombinant IgG Abs

Recombinant Abs were prepared from transiently transfected Expi293 cells as previously described (46, 47). Each recombinant Ig heavy- and light-chain plasmid pair was co-transfected at an equal ratio into Expi293 cells using ExpiFectamine (Invitrogen, Waltham, MA). After 17 hours of culture, enhancers provided with the kit were added to transfected Expi293 cells and incubated for five days at 37°C in 8% CO_2_. Cell culture supernatants were harvested and incubated with Pierce Protein A agarose beads (Invitrogen, Waltham, MA) overnight at 4°C on a rotating shaker. The beads were then collected in a chromatography column. Following PBS/NaCl wash, eluate was neutralized with trizma hydrochloride and then dialyzed against phosphate buffered saline (PBS) pH 7.4. Purified Abs were characterized by SDS-PAGE and Western blot analyses under both reducing and nonreducing conditions (39, 46, 48).

### UBE3A binding assay

A Luminex bead-based assay was used to determine the binding activity of Abs to host protein UBE3A, as previously described (49, 50). Briefly, 5×10^6^ carboxylated beads (regions 15, 17, 24, 53, 72 [Luminex Corp, Austin, TX]) were covalently linked to 25 μg of the following antigens: for goat-anti-human-kappa (Southern Biotech, Birmingham, AL), goat-anti-human-lambda (Southern Biotech), UBE34 (Abcam, Cambridge, MA), BSA [Sigma] and goat-anti-human-IgG (Fc) (Jackson ImmunoResearch, West Grove, PA), respectively. Starting at a 2 µg/ml concentration, recombinant Abs were serially diluted at a 1:3 ratio with washing buffer (PBS containing 0.05% NaN_3_, 0.05% Tween 20, 1% BSA and 1% milk). The five antigen-coupled microsphere bead mixtures were incubated with diluted Abs (with VRC01 used as a positive control, and 151K as a negative control), for 2 hours at room temperature (RT) in 96-well filter bottom plates (MilliporeSigma, Burlington, MA) using mild agitation. Beads were then washed 3 times, after which they were incubated an hour further with 2 µg/ml PE goat anti-human IgG (Southern Biotech), again using mild agitation. After three washes, beads were resuspended in assay buffer and fluorescence signals from each microsphere was measured on a Bio-Plex 3D Suspension Array System (Bio-Rad Laboratories, Hercules, CA).

### Host protein binding assay

Binding of recombinant CH103 Ab mutants to 9,400 human proteins was determined using a ProtoArray (Invitrogen, Waltham, MA) in duplicate, according to the manufacturer instructions and as described previously (13, 23, 50, 51). In brief, protein microarrays were blocked with 2 µg/ml of recombinant Abs or 151K, an isotype-matched (IgG1, κ) human myeloma control Ab (Southern Biotech) for 1.5 hours at 4°C. Ab binding to array proteins was detected using 1 µg/ml of Alexa Fluor 647-conjugated anti-human IgG (Invitrogen), using mild agitation at 4°C for 1.5 hours. Microarrays were scanned using a GenePix 4000B scanner (Molecular Devices, San Jose, CA). Fluorescence intensities of Ab binding to each protein were quantified by aligning image data with the GenePix Pro 5.0 program (Molecular Devices) using lot-specific protein spot definitions provided by the manufacturer, and by comparing Ab-binding patterns to the 151K control.

### HEp-2 cell staining

All Abs were assayed for poly-/autoreactivity to HEp-2 cells by indirect immunofluorescence staining as reported previously (16, 51), with minor modifications. Briefly, HEp-2 cell slides were incubated with 50µg/mL Abs (in a 20 µl volume) onto individual predetermined spots on slide surfaces, with 2F5 and CH106 bnAbs as positive controls, and 17B, and CH103_UCA Abs as negative controls. After incubation for 25 minutes at RT, three washes, and addition of 0.4 µg of secondary goat anti-human IgG-FITC Ab (Southern Biotech) in 20 µl, slides were incubated in a humid chamber for 25 minutes in the dark. Finally, after washing and drying, a drop of 50% glycerol was added to each spot, and slides were covered with 24x60-mm coverslips. Images were taken on an Olympus AX70 microscope.

### Determination of poly-/autoreactivity by ELISA

Binding activities of Abs to nucleic acids and host proteins were determined by ELISA, based on previously described methods (52). Briefly, antigens were coated at an optimal concentration of 20µg/ml in Costar 3700 plates (Corning Inc., Corning, NY) overnight at 4°C and plates were blocked for 1h at RT. Abs were tested using the same lots of each antigen, in serial dilutions for 45 min at RT. For DNA ELISAs, plates were coated with 20 µg/ml poly-lysine (MilliporeSigma) for 1h at RT, washed 3 times with PBS and blocked with PBS containing 3% BSA for 1h at RT. After three washes, DNA in saline sodium citrate buffer was added for 1h, washed further, and incubated in Abs for 45 min. HRP-conjugated human IgG secondary Ab was added at the optimal dilution of 1:30,000 in PBS (containing 0.05% Tween-20 and 1% BSA) for 30 min and developed using TMB substrate. Plates were read at 450 nm in a SpectraMax 384 PLUS reader (Molecular Devices, San Jose, CA). Results were reported as logarithmic area under the curve (LogAUC), unless otherwise noted.

### Structural analysis

To visualize locations of mutations in the CH103 lineage Abs, the structure of CH103 in the complex with gp120 (PGB ID: 4JAN) was used to determine their potential impacts using the Swiss-Model server (https://swissmodel.expasy.org/). Predicted structural models generated were then further analyzed using PyMoL software (https://pymol.org/2/support.html?). Surface electrostatic potentials were visualized with the assistance of the APBS plugin (52).

### Statistical analysis

The statistical analysis was performed using the SPSS version 26. The comparison between wild type and mutant antibodies were done with Mann-Whitney U test. The p values <0.05 were considered significant.

## Results

### Individual mutations in HCDR2 increase poly-/autoreactivity at the late maturation stage of the CH103-106 bnAb lineage

**Figure 1:**
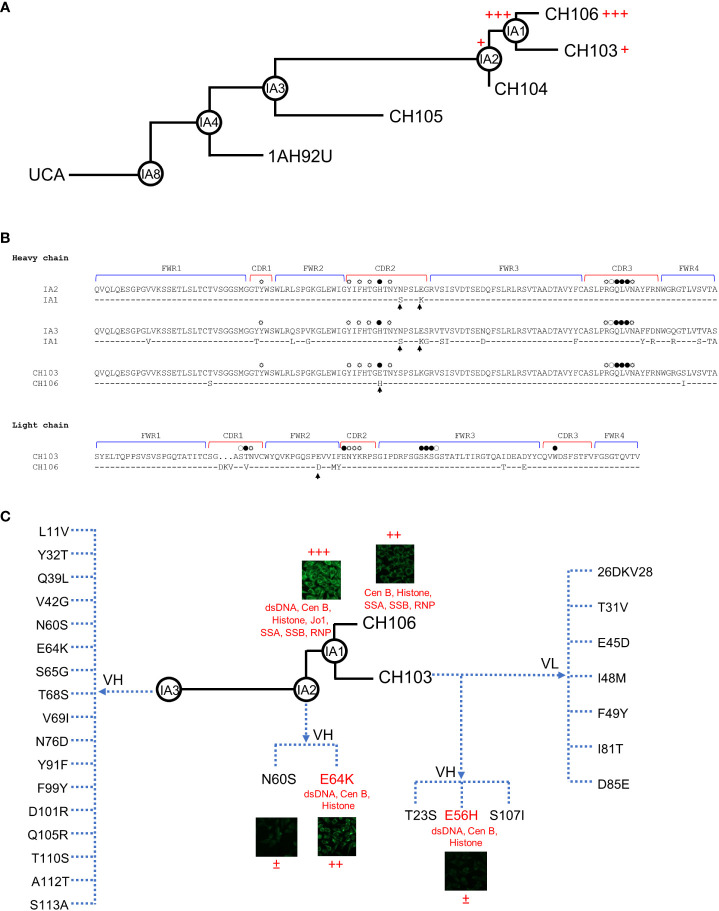
Increased poly-/autoreactivity by single mutations in CH103 lineage bnAbs. **(A)** Development of poly-/autoreactivity during the maturation process of later CH103-106 bnAb clonal lineage members. Inferred evolutionary intermediate Abs are shown in circles and the levels of poly-/autoreactivity are indicated by numbers of plus signs. **(B)** Alignment of AA sequences of the CH103-106 bnAb lineage pairs being compared in this study, with complimentary determinant regions (CDR) and framework regions (FWR) indicated. AAs that bind to HIV-1 Env through main chains, side chain, and both chains are indicated by open, spiked open, and solid circles, respectively. AA substitutions that lead to poly-/autoreactivity are indicated by arrows. **(C)** Poly-/autoreactivity of CH103-106 lineage Ab mutants. Poly-/autoreactivities of CH103-106 lineage mutant Abs were determined by HEp-2 staining and Athena autoantigen assays. Each test was done in duplicates and similar results were observed in repeated experiments. The mutations for IA3 are those absent in IA3 but present in IA1. The mutations for CH103 are those different between CH103 and CH106 and changed to the amino acids present in CH106. ±: weak, <10% positive cells; +: intermediate, 10%-30% positive cells; ++: strong, 31%-70% positive cells; +++: stronger, >70% cells.

Previously, we reported no detectable poly-/autoreactivity for the intermediate IA3 of the CH103-106 bnAb lineage, weak poly-/autoreactivity for the later intermediate IA2 and a mature bnAb CH103, and strong poly-/autoreactivity for the latest intermediate IA1 and another mature bnAb CH106 [**Figure 1A** and (13)]. To further understand the role of mutations acquired during the late maturation stage of the CH103-106 bnAb lineage, we performed comprehensive *in vitro* mutagenesis of these late intermediates and mature bnAbs, in an effort to dissect the impact of their individual changes on neutralization and poly-/autoreactivity. We started our mutagenesis analysis with the IA2 and IA1 pair of intermediates because they differ from each other by only two amino acids (aa) in the heavy chain complementarity determining region 2 (HCDR2) within the variable gene segment (V_H_), but not at sites contacting the HIV-1 gp120 glycoprotein (**Figure 1B**). Furthermore, both IA2 and IA1 share the same light chain (13), but IA2 only shows weak poly-/autoreactivity compared to the more mature IA1. Thus, to understand if either of the two mutations in IA1 are responsible for its increased poly-/autoreactivity, we introduced the N60S and E64K mutations individually into IA2. We first assayed the two mutant IA2 Abs for their ability to bind to a human epithelial line, HEp-2 (**Figure 1C** and **S1**). We observed that IA2 was negative for binding while IA1 was strongly positive, as previously reported (13). However, the IA2_E64K mutant was strongly positive, albeit less so than IA1, while the IA2_N60S mutant only showed weak positive staining (**Figure 1C**). Autoantigen ELISA analysis confirmed that the IA2_E64K mutant was reactive to DNA and some host proteins, but less so than IA1, while the IA2_N60S mutant lacked all reactivity to DNA, RNA or host proteins (**Figure 1C** and **Table S1**). These results thus show that the E64K mutation accounts for the majority of the increased poly-/autoreactivity exhibited by IA1, but that the full poly-/autoreactivity of IA1 requires both mutations.

To further investigate which other mutations could be involved in specifying poly-/autoreactivity before the later intermediate IA2 branchpoint of the CH103-106 bnAb lineage, we extended our mutagenesis analysis to comparisons between 1A1 and the significantly less mature and non-polyreactive intermediate IA3, with the latter differing from IA1 by 17 AAs in its V_H_ region (**Figure 1B**). After systematically introducing each of these 17 mutations into IA3 to examine if any could individually render IA3 poly-/autoreactive, none were found to react to HEp-2 or autoantigens (**Figure S1** and **Table S1**). Intriguingly, the E64K mutation that rendered IA2 strongly poly-/autoreactive did not increase poly-/autoreactivity of IA3. These results therefore indicate that mutations other than the E64K are also required for development of both poly-/autoreactivities during the maturation of the CH103-106 lineage. This is consistent with the notion that multiple mutations develop between IA3 and IA2 to generate a poly-/autoreactivity pocket, but that the E64K mutation is one of the culminating changes and breaks the threshold leading to IA1’s much higher polyreactivity.

To also examine whether yet other mutations, acquired even later than the IA1 branchpoint, could affect poly-/autoreactivity, we extended our mutagenesis analysis to the latest known SHM events acquired in the CH103-106 lineage: those found in the bnAbs CH103 and CH106, themselves. Notwithstanding the fact that both bnAbs have comparable SHM levels that contribute to reaching high affinity to HIV-1 and neutralization, only CH106 is significantly autoreactive, while CH103 is only slightly autoreactive (13). This represented a unique opportunity to investigate if mutations govern autoreactivity and broad neutralization independently. CH106 is different from CH103 by 10 aa differences: three in the V_H_ chain (T23S, E56H, and S107I) and seven differences in the V_L_ chain (T31V, E45D, I48M, F49Y, I81T, D85E, and a 3-aa (DKV) insertion) (**Figure 1B**). To determine if any of these ten differences specified CH106’s higher poly-/autoreactivity levels, we introduced each different amino acid individually into CH103. Only the E56H substitution in the HCDR2 region rendered CH103 positive for HEp-2 staining (**Figures 1C** and **S1**) as well as reactive to dsDNA, Cen B and Histone in autoantigen ELISA assays (**Figure 1C** and **Table S1**). None of the other eight aa substitutions (nor the 3-aa insertion) tested in either assay rendered CH103 more poly-/autoreactive. Thus, it appears that by replacing the glutamic acid with a histidine acid at position 56, CH103 gained some autoreactivity while maintaining broad neutralization. It is well known that the poly-/autoreactivity test results vary between different methods since they do not react to the same targets. Thus, it is important to use different methods to have a better understanding of poly-/autoreactivity of tested antibodies in order to avoid biases by each assay. The poly-/autoreactivity of test results were generally consistent among different assays in our study, although there were some minor discrepancies (**Table S3**).

### The E64K mutation elevates overall poly-/autoreactivity to host proteins

**Figure 2:**
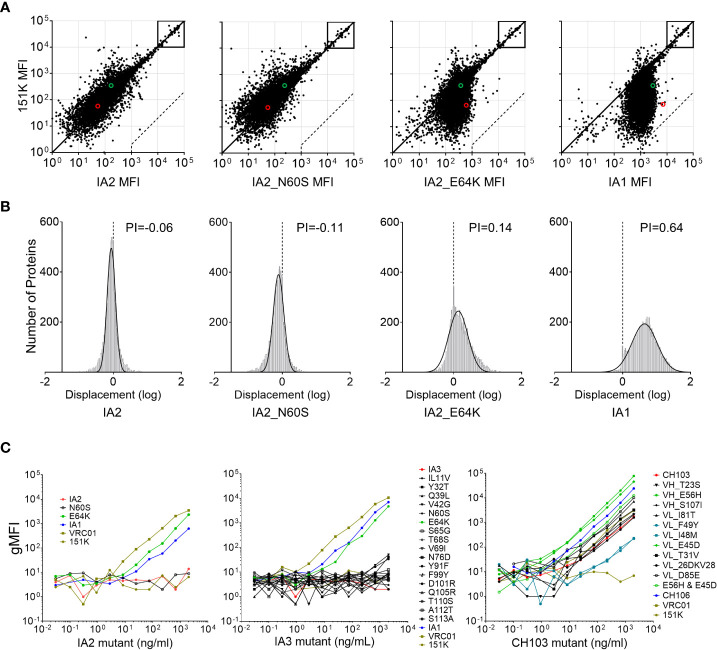
CH103-106 lineage Ab mutant reactivities to host proteins. **(A)** Determination of host protein binding by protein array analysis. Binding of CH103-106 lineage Abs to 9,400 human proteins was measured using ProtoArrays and compared to a control Ab, 151K. Diagonals in each graph represent equal fluorescence intensities (equivalent binding) by CH103-106 lineage Abs and 151K control Ab. Boxes indicate the internal controls used to ensure loading of Abs and secondary detection reagents resulted in equal binding by Ab pairs. Dashed lines indicate the 500-fold signal/background ratio defined as the cutoff for autoreactivity. The binding to UBE3A and BHMT2 is indicated by the red circle and green circle separately. **(B)** Frequency histograms of protein displacement. The protein displacement (log) from the diagonal line by CH103-106 lineage Abs, compared to the control Ab, 151K are shown. Negative displacements indicate stronger binding by the negative control 151K. The Gaussian mean of all array protein displacements is termed the polyreactivity index (PI). **(C)** Protein UBE3A binding analysis. UBE3A binding activity was determined by measuring Mean Fluorescence Intensity (dMFI) for each of the IA2, IA3 and CH103 mutant Ab sets. For each Ab set, both parental Abs from the CH103-106 lineage’s late intermediate Ab or bnAb pair being compared are included for comparison, as is the positive control (VRC01) and the negative control (151K).

Only two mutations appeared to be selectively responsible for increased poly-/autoreactivities between IA2 and IA1 by the Athena autoantigen assay test and the Hep-2 staining method: the E64K mutation (which caused much higher levels of poly-/autoreactivity in IA2) and the N60S mutation (which led to only a marginal increase). To further investigate the reactivities of each mutant to host proteins, we measured their binding to a protein microarray displaying 9,400 human proteins, using the polyreactivity index (PI), which plots the distance of each data point from the diagonal as a frequency histogram and shows the Gaussian mean of the distances from the diagonal, control Ab 151K (**Figures 2A, B**). When compared to the 151K which only binds the human betaine-homocysteine methyltransferase 2 (BHMT2) protein, IA2 showed a comparable binding pattern (PI = -0.06), indicating no increased binding to host proteins (**Figure 2B**). In stark contrast, IA1 avidly increased its binding ability to the majority of host proteins (PI = 0.64), confirming its strong poly-/autoreactivity (**Figures 2A, B**). The IA2_E64K mutant, while exhibiting detectable binding to most host proteins, had an overall binding pattern which did not shift over as far from the diagonal as did IA1 (PI = 0.14), indicating that it increases IA2’s polyreactivity, albeit not fully. The N60S mutant, meanwhile had a binding pattern more similar to that of the 151K control, thus confirming its minimal poly-/autoreactivity (**Figures 2A, B**). These results further confirm that while the E64K mutation plays a critical role in increasing the poly-/autoreactivity of CH103-106 lineage Abs, both the E64K and N60S mutations are required to reach the full level of poly-/autoreactivity seen with IA1.

### Impact of mutations on binding to the host protein UBE3A

The protein microarray analysis of IA1, IA2 and the two IA2 mutants showed that Abs with the E64K change bound to UBE3A at nearly 10-fold higher affinity than Abs without it (**Figure 2A**; indicated by red circles). CH103 was previously found to avidly bind the host protein UBE3A and binding kinetics were characterized using a well-validated Luminex bead assay (13). Thus, we next sought to use this same assay to determine the binding kinetics of the E64K mutant and if any other mutants that we generated could render their respective parental Abs more reactive to UBE3A. Amongst the two IA2 mutants tested, the E64K mutant bound UBE3A well and was similar as IA1 (p=0.347), but both bound to UBE3A at lower affinity than the positive control, VRC01 (**Figure 2C**, left panel). In contrast, the N60S mutant, like IA2 and the negative control 151K, showed no reactivity to UBE3A. When all 17 IA3 mutants were tested, only the E64K mutant showed a comparable, high level of UBE3A reactivity as IA1 (p=0.602), while no other mutants bound to UBE3A (**Figure 2C**, middle panel). Finally, with respect to the 11 CH103 mutants, both the V_H_ E56H mutant and the V_L_ E45D mutant were significantly more reactive to UBE3A than the parental CH103 bnAbs (p=0.028 and p=0.047, respectively) (**Figure 2C**, right panel). However, when we tested a CH103 mutant containing both the E56H and E45D mutations (as both are present in the parental CH106 bnAb), its reactivity to UBE3A was similar as the parental CH106 (**Figure 2C**, right panel). The V_L_ F49Y or I48M mutant reduced CH103’s binding ability to UBE3A by about 10-fold (p=0.016), while all other CH103 mutants had binding levels akin to parental CH103 (**Figure 2C**, right panel). Collectively, these results clearly demonstrate that individual mutations in CH103 have varying impacts on its ability to bind UBE3A.

### Mutations associated with poly-/autoreactivity have no impact on neutralization potency and breadth

**Figure 3:**
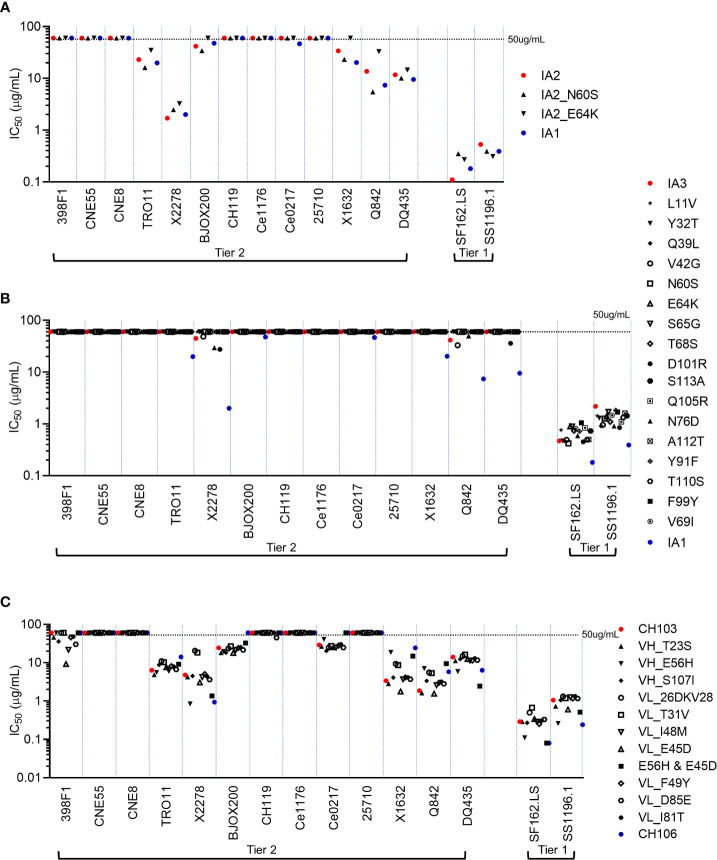
Neutralization analysis of CH103-106 lineage Ab mutants. Neutralization potencies and breadths were determined for IA2 mutant Abs **(A)**, IA3 mutant Abs **(B)** and CH103 mutant Abs **(C)**. IC_50_ was determined for each Ab against 13 tier-2 HIV-1 Env-pseudoviruses and two tier-1 HIV-1 Env pseudoviruses on TZM-bl cells. Each test was done in duplicates and similar results were observed in repeated experiments. Dashed lines represent the highest Ab concentration (50μg/ml) used in the experiment.

The temporal relationship between when neutralization breadth is initially seen (IA2) and polyreactivity peaks (IA1) suggests that while many mutations might contribute to both outcomes, only 3 aa changes, all in HCDR2, selectively impact polyreactivity: E64K and N60S (acquired in 1A1) and E56H (between the two mature bnAbs). To formally determine if any of the individual mutant Abs we generated had altered neutralization potency and/or breadth, we tested their neutralization activity against two easy-to-neutralize tier-1 HIV-1 Env pseudoviruses and 13 hard-to-neutralize tier-2 HIV-1 Env pseudoviruses (including 11 global panel viruses (398F1, CNE55, CNE8, TRO11, X2278, BJOX200, CH119, Ce1176, Ce0217, 25710 and X1632) (44). Both N60S and E64K mutants of IA2 had similar neutralization potencies and breadth as parental IA1 and IA2 (which themselves, have comparable overall neutralization profiles; **Figure 3A** and **Table S2**). Among the series of 17 mutants of IA3, we found that the vast majority had similar neutralization profiles to parental IA3 and lacked neutralization activity against most of the tier-2 viruses (**Figure 3B** and **Table S2**). All these changes were small and not statistically significant.

Finally, amongst the 10 CH103 mutants that each contained the amino acid found in CH106 at the same position, each only had marginal impact on neutralization potency, with nearly all changes in neutralization potency within a 0.5 log IC_50_ (**Figure 3C** and **Table S2**). The only exception was that some mutations made CH103 capable of neutralizing 398F1, albeit weakly. Taken together, the individual mutations that we introduced into either the two intermediates or the mature CH103 lineage Abs had only little or no impact on neutralization potency and breadth, relative to their respective parental Abs. Most importantly, mutations which significantly increase poly-/autoreactivity also have no impact on neutralization activity.

### Structure modeling analysis of the mutations associated with increased poly-/autoreactivity

**Figure 4:**
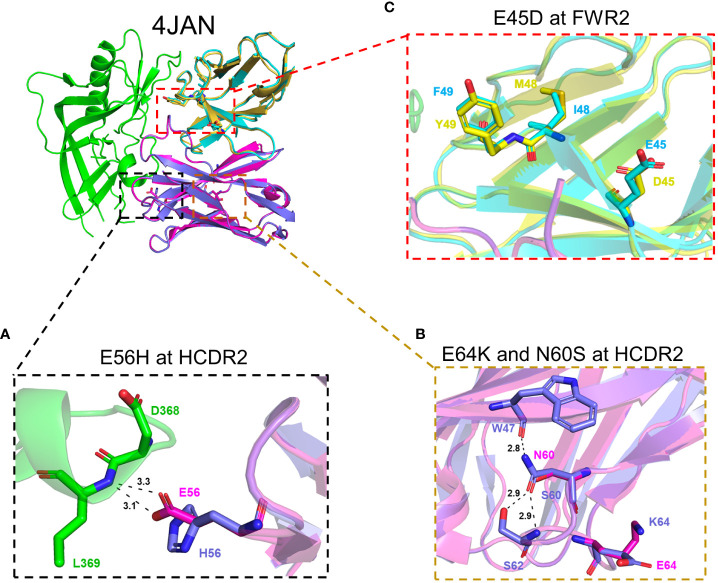
Structural analysis of mutations responsible for poly-/autoreactivity. Shown are the potential changes in interactions between relevant aa residues in Env and mutations that increase poly-/autoreactivity in HCDR2 (E56H) of the V_H_ chain between CH103 and CH106 **(A)**, in HCDR2 (E64K and N60S) of the V_H_ chain between IA2 and IA1 **(B)** and in FRW2 (E45D) of the V_L_, chain between CH103 and CH106 **(C)**. The HIV-1 gp120 protein is depicted in green and the CH103 heavy chain and light chain are shown in magenta and cyan, respectively.

To visualize how these mutations (accumulated during the latter developmental stages of this clonal bnAb^+^ B-cell lineage) impact poly-/autoreactivity and neutralization ability, we modeled their interactions with the HIV-1 Env, based on the known co-crystal structure of Env in complex with CH103 (13). All but one change (E56H in the V_H_ of CH103) were not at any Env-CH103 contact sites (**Figure 1B**). Thus, neutralization ability is not expected to be changed by those mutations. At position 56 in V_H_, glutamic acid (E) and histidine (H) are very similar in size, but the E56H substitution may cause side-chain changes of D368 in Env and form more stable hydrogen bonds with Env for more stable binding (**Figure 4A**). Although glutamic acid and histidine are differently charged, both CH103 (with E56) and CH106 (with H56) have comparable neutralization activity against both tier-2 and tier-1 viruses (changes within 1 log IC_50_; **Figure 3C**). These results show that the glutamic acid and histidine substitution between CH103 and CH106 at position 56 in V_H_ also doesn’t affect neutralization ability.

Since only four of the 29 total mutants during late B-cell lineage development rendered their respective parental Abs more poly-/autoreactive (N60S, E64K and E56H in the HCDR2; and E45D in the framework region-2; **Figure 1B**), we focused on further modeling these specific four mutants. Intriguingly, with respect to the three introduced HCDR2 mutants, two of them (E64K in IA2 and E56H in CH103), rendered their respective parental Abs reactive to Hep-2 cells, multiple autoantigens (dsDNA, Cen B and Histone) and the host protein UBE3A (**Figures 1C**, **2C**). Both these HCDR2 mutants changed a negative-charged glutamic acid (E) to a positive-charge amino acid (K or H), reminiscent of previous reports in which positive charge-conferring mutations at distinct residues within the same HCDR2 additively contribute to polyreactive potency of auto-Abs (53–55). Thus, unlike for neutralization activity, net charge differences among substituted residues (especially for E56H in CH103, which is at the Env-CH103 contact site) may play an important role in increased poly-/autoreactivities of the CH103 lineage Abs.

Finally, two other mutations (N60S in CDR2 of V_H_ and E45D in FWR2 of V_L_) only rendered their parental Abs weakly more poly-/autoreactive; the N60S mutation rendered IA2 weakly reactive to HEp-2 cells and the E45D mutation rendered IA3 highly reactive to UBE3A. Both are not at the Env-CH103 interface (**Figures 1B**, **4**). However, the sizes and charges between the parental and substituted residues are very similar at both sites (**Figures 4B, C**). How such substitutions (with similar aa residues) can still lead to alterations in reactivity to autoantigens or host proteins warrants further investigation.

## Discussion

In this study, we systematically investigated the impact of mutations accumulated during late stages of the maturation process of the CH103-106 lineage bnAbs. We find that the key mutations that render this lineage’s Abs poly-/autoreactive had little impact on neutralization potency and breadth. This formally demonstrates, for the first time (to our knowledge) that the potency and breadth of neutralization and poly-/autoreactivity do not necessarily have to be governed by the same mutations during late maturation of an HIV bnAb and thus decoupling of autoreactivity and neutralization can be achieved during the induction of protective anti-HIV Ab responses.

Among a total of 29 mutations from three sets of compared Abs (IA2 vs. IA1, IA3 vs. IA1 and CH103 vs. CH106), four (E64K, E56H, N60S and E45D) of them rendered the Abs poly-/autoreactive. Three (E64K, E56H and N60S) were found in HCDR2 and one (E45D) was in FWR2 of V_L_. Among them, only the E56H mutation was found at the Env-Ab interface. Since both the E64K mutation in IA2 and the E56H mutation in CH103 changed a negatively charged residue to a positively charged one, these changes likely allowed IA2 and CH103 to bind to autoantigens and host proteins in multiple assays (Hep-2 staining, Athena autoantigen assay and UBE3A binding assay). This is consistent with several previous studies reporting the promotion of polyreactivity in auto-Abs by positively charged residues in HCDR2 (53–55). Also consistent with this notion is that in CH103, the natural mutation removed the histidine at position 56 (and replaced it with the negatively charged glutamic acid while retaining the E64K mutation). This further supports the notion that mitigation of autoreactivity, can be accomplished while keeping neutralization breadth. Therefore, it is likely that CH103 accomplished a reduction in the net positive charge of CDR2 and that the natural evolution of CH103 resulted in a decoupling of autoreactivity and neutralization. This represents a novel finding that can be used to guide novel vaccine approaches or modifications of pre-existing ones.

The N60S mutation in IA2 and the E45D mutation in CH103 marginally increased autoreactivity. How such conservative amino acid substitutions can still lead to alterations in reactivity to autoantigens or host proteins warrants further investigation but the marginal impact is consistent with these being changes between amino acids of similar properties in terms of charge and size. The totality of these data indicates that non-conservative amino acid changes, particularly those that result in a different charge and at HCDR2 play an important role in modulating poly-/autoreactivity during bnAb CH103 lineage maturation. Interestingly, a recent series of studies examining the relationship of self-reactivity and glycan-dependent binding of a bnAb to the HA stalk of another highly variable pathogen, Influenza, was also found to have HCDR2 residues crucial for controlling its self-reactivity (56–60). This raises the intriguing possibility that HCDR2 residues like those identified in this study (i.e., those *selectively* governing CH103’s self-reactivity but not its neutralization specificity) may also exist in not only other HIV-1 bnAb lineages (induced to either the CD4bs or alternate Env vaccine targets) but even in those generated to other highly-mutable human pathogens. Such lineages would be prime candidates to re-design vaccine protocols that incorporate immunogens selected for, based on their ability to more selectively bind bnAb epitopes.

Another intriguing aspect of our study is that introduction of the E64K mutation to an earlier intermediate IA3 did not alone accomplish full autoreactivity, indicating at least some of the subsequent multiple mutations seem required for both neutralization and polyreactivity in an incremental fashion. This suggests a model that a carefully evolved antigen-binding pocket requiring multiple changes to increase neutralization leaves the B cell susceptible to autoreactivity, with the E64K mutation pushing the antigen binding pocket over the autoreactivity threshold. While this complicates a putative decoupling strategy since it suggests the cumulative impact of earlier mutations may set the stage for both autoreactivity and neutralization, the identification of single changes as catalysts for autoreactivity but not neutralization once the pocket is established, suggests that late maturation strategies can be used to drive the response away from autoreactivity and in favor of neutralization. Indeed, CH103 appears to have resolved this conundrum by reducing the pocket net positive charge by removing a positively charged histidine and replacing it with a negatively charged glutamic acid elsewhere in CDR2 (position 56). Some mutations have been found to simultaneously enhance neutralization and polyreactivity for bnAbs targeting the CD4bs and MPER epitopes (61, 62). Consistent with this, previous studies also show that substitutions by positively charged residues in CDR regions are likely to lead to increased poly-/autoreactivity (55, 63–65). Thus, from the cumulative data presented here and previous studies, we propose that mutations inducing a charged HCDR2 may predict poly-/autoreactivity and potentially inform design of immunogens that can dissuade such mutations from occurring or promoting those that reduce autoreactivity, particularly in later stages of the response (i.e. during boosting).

The similar neutralizing activity but different poly-/autoreactivity between CH103 and CH106 antibodies also indicate that the outset of the neutralization and poly-/autoreactivity can be decoupled during the maturation of the CH103-106 lineage. Here we clearly defined the sequence-related details of the two parameters in the CH103-106 lineage. However, whether the neutralizing activity and poly-/autoreactivity can be similarly decoupled in other bnAbs targeting CD4bs or other sites requires further investigation.

These observations have important implications in HIV-1 vaccine development. For example, it is thought that residual poly-/autoreactive B cell clones are generally at a survival disadvantage and require unusually strong BCR and CD4 T-cell signals to overcome their inactivation and generate durable Ab responses (37, 38). Combined with the tendency of these autoreactivity-specifying mutations to be positively charged, and coupled with this study’s central finding that at least some of these residues in bnAbs do not overlap with foreign antigen specificity (i.e., for bnAbs specifically, this means their binding to Env from a wide array of HIV quasi-species), it may now be possible to design novel vaccines that can induce potent and broad neutralization activity without triggering tolerizing autoreactivity that would generally disfavor B cell development and maturation to full breadth/potency. For example, panels of candidate immunogens could be initially engineered and iteratively improved upon, by repeatedly screening top candidates in each round, against the CH103 lineage nbAb mutants generated in this study. In this regard, this process of ‘redeeming’ bnAbs from tolerance mechanisms through vaccination has been elegantly demonstrated in studies examining the degree of self vs foreign antigen overlap among HCDR2 residues, using a hierarchy of HEL immunogens and Ig ‘knock-in’ (KI) models expressing anti-HEL Abs (66–69). Currently, ongoing studies involving immunization of CH103 UCA H+L KI mice [a vaccine model we designed to express the CH103 lineage unmutated common ancestral Ab (70); reviewed in (71)] with a multimerized trimeric Env-based vaccine protocol, we have already identified two independently derived, vaccine-induced CH103-106-like nAb lineages that demonstrate heavy selection pressure for relevant combinations of these exact same ‘decoupling’ HCDR2 residues (Leaman, Verkoczy, Zwick et al., personal communication). We thus anticipate that ‘redeemed’ CH103 oligoclonal families of mutated memory IgG^+^ clones (harboring the same or similar de-coupling HCDR2 mutations as we identified in this study) can be reproducibly elicited by appropriate vaccination. However, given the complexity of the maturation pathway seen in the original CH103 lineage in a HIV-1 chronically infected subject, we expect such clones will continue to occur infrequently with our ‘early generation’ CH103-like vaccine regimens. However, with a now clear identification of amino acid targets to favor and disfavor, iterative immunogen design strategies as those described above may represent the next research frontier needed to achieve effective HIV vaccine protocols.

We thus anticipate that ‘redeemed’ CH103 oligoclonal families of mutated memory IgG+ clones (harboring the same or similar de-coupling HCDR2 mutations as we identified in this study) can be reproducibly elicited by appropriate vaccination. However, given the complexity of the maturation pathway seen in the original CH103 lineage in a HIV-1 chronically infected subject, we expect such clones will continue to occur infrequently with our ‘early generation’ CH103-like vaccine regimens. In addition, the immunogenicity impact of these mutations will have to be determined in future studies to select the best pathways to induce through vaccination.

## Data availability statement

The original contributions presented in the study are included in the article/**Supplementary Material**. Further inquiries can be directed to the corresponding authors.

## Author contributions

FG and LV conceived, designed and supervised the study. XL and DL performed experiments and provided key reagents. ZL and JL performed structure analysis and evaluated all data. XL, DL, MD, LV and FG analyzed data. XL, MD, LV, and FG wrote the manuscript. All authors contributed to the article and approved the submitted version.

## Funding

This work was supported by the National Key Research and Development Program of China (2021YFC2301500), as well as by NIH R01 grants AI087202 (to LV) and AI118571 (to LV and FG) from NIAID.

## Acknowledgments

We thank M. Anthony Moody, Robert Parks and Barton F. Haynes for performing auto-Ab assays and Garnett H. Kelsoe for helpful discussions regarding UBE3A binding analysis.

## Conflict of interest

The authors declare that the research was conducted in the absence of any commercial or financial relationships that could be construed as a potential conflict of interest.

## Publisher’s note

All claims expressed in this article are solely those of the authors and do not necessarily represent those of their affiliated organizations, or those of the publisher, the editors and the reviewers. Any product that may be evaluated in this article, or claim that may be made by its manufacturer, is not guaranteed or endorsed by the publisher.
