# Supplementary material for: Autoreactivity and broad neutralization of antibodies against HIV-1 are governed by distinct mutations: Implications for vaccine design strategies
Source: Front Immunol. 2022 Nov 18;13:977630. doi: 10.3389/fimmu.2022.977630 (PMC9720396; doi:10.3389/fimmu.2022.977630)
Supplement: Supplementary file 1 [file Presentation_1.pdf]

**Figure S1**

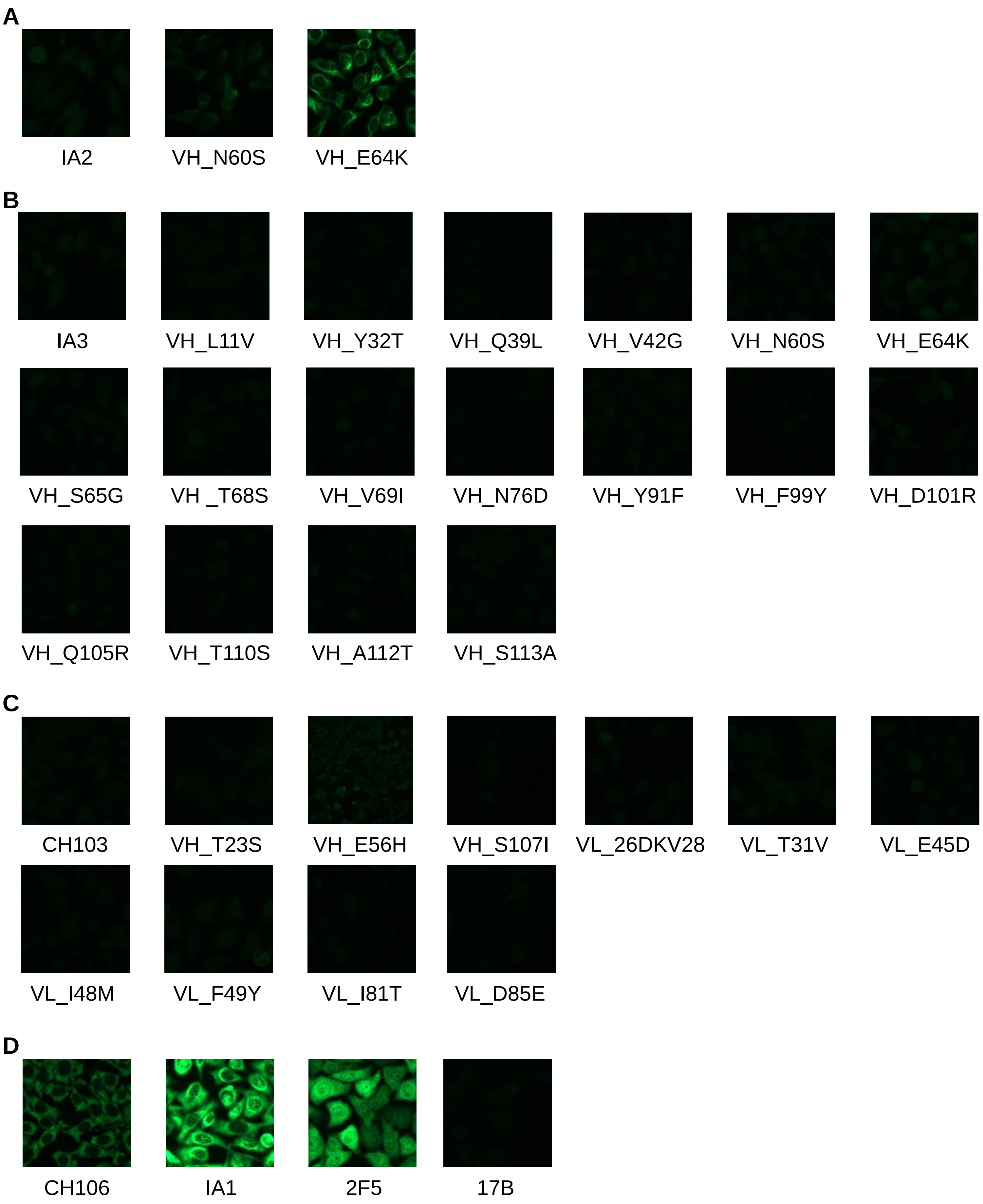

A
IA2
VH_N60S
VH_E64K
B
IA3
VH_L11V
VH_Y32T
VH_Q39L
VH_V42G
VH_N60S
VH_E64K
VH_S65G
VH _T68S
VH_V69I
VH_N76D
VH_Y91F
VH_F99Y
VH_D101R
VH_Q105R
VH_T110S
VH_A112T
VH_S113A
C
CH103
VH_T23S
VH_E56H
VH_S107I
VL_26DKV28
VL_T31V
VL_E45D
VL_I48M
VL_F49Y
VL_I81T
VL_D85E
D
CH106
IA1
2F5
17B

## Table S1. Reactivity to autoantigens by the CH103 lineage Ab mutants

| Antibody | | AutoAg ELISA | | | | | | | | |
|---|---|---|---|---|---|---|---|---|---|---|
| | | DNA | CenB | Hisatone | Jo-1 | SSA | SSB | Scl-70 | Sm | RNP |
| IA2 abs | IA2 | - | - | - | - | - | - | - | - | - |
| | IA2_N60S | - | - | - | - | - | - | - | - | - |
| | IA2_E64K | + | + | + | - | - | - | - | - | - |
| | | | | | | | | | | |
| IA3 abs | IA3 | - | - | - | - | - | - | - | - | - |
| | IA3_L11V | - | - | - | - | - | - | - | - | - |
| | IA3_Y32T | - | - | - | - | - | - | - | - | - |
| | IA3_Q39L | - | - | - | - | - | - | - | - | - |
| | IA3_V42G | - | - | - | - | - | - | - | - | - |
| | IA3_N60S | - | - | - | - | - | - | - | - | - |
| | IA3_E64K | - | - | - | - | - | - | - | - | - |
| | IA3_S65G | - | - | - | - | - | - | - | - | - |
| | IA3_T68S | - | - | - | - | - | - | - | - | - |
| | IA3_V69I | - | - | - | - | - | - | - | - | - |
| | IA3_N76D | - | - | - | - | - | - | - | - | - |
| | IA3_Y91F | - | - | - | - | - | - | - | - | - |
| | IA3_F99Y | - | - | - | - | - | - | - | - | - |
| | IA3_D101R | - | - | - | - | - | - | - | - | - |
| | IA3_Q105R | - | - | - | - | - | - | - | - | - |
| | IA3_T110S | - | - | - | - | - | - | - | - | - |
| | IA3_A112T | - | - | - | - | - | - | - | - | - |
| | IA3_S113A | - | - | - | - | - | - | - | - | - |
| | | | | | | | | | | |
| CH103 abs | CH103 | - | - | - | - | - | - | - | - | - |
| | CH103 VH_T23S | - | - | - | - | - | - | - | - | - |
| | CH103 VH_E56H | + | + | + | - | - | - | - | - | - |
| | CH103 VH_S107I | - | - | - | - | - | - | - | - | - |
| | H103 VL_26DKV28 | - | - | - | - | - | - | - | - | - |
| | CH103 VL_T31V | - | - | - | - | - | - | - | - | - |
| | CH103 VL_E45D | - | - | - | - | - | - | - | - | - |
| | CH103 VL_I48M | - | - | - | - | - | - | - | - | - |
| | CH103 VL_F49Y | - | - | - | - | - | - | - | - | - |
| | CH103 VL_I81T | - | - | - | - | - | - | - | - | - |
| | CH103 VL_D85E | - | - | - | - | - | - | - | - | - |
| | | | | | | | | | | |
| Control abs | IA1 | + | + | + | + | + | + | - | - | + |
| | CH106 | - | + | + | - | + | + | - | - | + |
| | 17B | - | - | - | - | - | - | - | - | - |
| | 2F5 | - | - | - | - | - | - | - | - | - |

Sjogren’s syndrome antigens A and B (SSA and SSB, respectively), Smith antigen (Sm), ribonucleoprotein (RNP), centromere B (Cen B), histone, scleroderma 70 (Scl-70) and Jo-1 proteins, and dsDNA,

# Table S2. Neutralization activity of the CH103 lineage Ab mutants against 15 HIV-1 Env-pseudoviruses.

| Virus ID | Clade | IA2 | IA2_N 60S | IA2_E 64K | IA3 | IA3_ L11V | IA3_Y3 2T | IA3_Q 39L | IA3_V 42G | IA3_N 60S | IA3_E 64K | IA3_S 65G | IA3_T6 8S | IA3_V 69I | IA3_N 76D | IA3_Y9 1F | IA3_F 99Y | IA3_D 101R |
|---|---|---|---|---|---|---|---|---|---|---|---|---|---|---|---|---|---|---|
| 398F1 | A | >50.00 | >50.00 | >50.00 | >50.00 | >50.00 | >50.00 | >50.00 | >50.00 | >50.00 | >50.00 | >50.00 | >50.00 | >50.00 | >50.00 | >50.00 | >50.00 | >50.00 |
| CNE55 | AE | >50.00 | >50.00 | >50.00 | >50.00 | >50.00 | >50.00 | >50.00 | >50.00 | >50.00 | >50.00 | >50.00 | >50.00 | >50.00 | >50.00 | >50.00 | >50.00 | >50.00 |
| CNE8 | AE | >50.00 | >50.00 | >50.00 | >50.00 | >50.00 | >50.00 | >50.00 | >50.00 | >50.00 | >50.00 | >50.00 | >50.00 | >50.00 | >50.00 | >50.00 | >50.00 | >50.00 |
| TRO11 | B | 23.07 | 16.15 | 35.03 | >50.00 | >50.00 | >50.00 | >50.00 | >50.00 | >50.00 | >50.00 | >50.00 | >50.00 | >50.00 | >50.00 | >50.00 | >50.00 | >50.00 |
| X2278 | B | 1.7 | 2.48 | 3.25 | 44.75 | >50.00 | >50.00 | >50.00 | 48.53 | >50.00 | >50.00 | >50.00 | >50.00 | >50.00 | 29.67 | >50.00 | >50.00 | 27.47 |
| BJOX200 | BC | 41.8 | 34.17 | >50.00 | >50.00 | >50.00 | >50.00 | >50.00 | >50.00 | >50.00 | >50.00 | >50.00 | >50.00 | >50.00 | >50.00 | >50.00 | >50.00 | >50.00 |
| CH119 | BC | >50.00 | >50.00 | >50.00 | >50.00 | >50.00 | >50.00 | >50.00 | >50.00 | >50.00 | >50.00 | >50.00 | >50.00 | >50.00 | >50.00 | >50.00 | >50.00 | >50.00 |
| Ce1176 | C | >50.00 | >50.00 | >50.00 | >50.00 | >50.00 | >50.00 | >50.00 | >50.00 | >50.00 | >50.00 | >50.00 | >50.00 | >50.00 | >50.00 | >50.00 | >50.00 | >50.00 |
| Ce0217 | C | >50.00 | >50.00 | >50.00 | >50.00 | >50.00 | >50.00 | >50.00 | >50.00 | >50.00 | >50.00 | >50.00 | >50.00 | >50.00 | >50.00 | >50.00 | >50.00 | >50.00 |
| 25710 | C | >50.00 | >50.00 | >50.00 | >50.00 | >50.00 | >50.00 | >50.00 | >50.00 | >50.00 | >50.00 | >50.00 | >50.00 | >50.00 | >50.00 | >50.00 | >50.00 | >50.00 |
| X1632 | G | 33.91 | 23.2 | >50.00 | >50.00 | >50.00 | >50.00 | >50.00 | >50.00 | >50.00 | >50.00 | >50.00 | >50.00 | >50.00 | >50.00 | >50.00 | >50.00 | >50.00 |
| Q842 | A | 13.65 | 5.49 | 33.04 | 41.55 | >50.00 | >50.00 | >50.00 | 33.09 | >50.00 | >50.00 | >50.00 | >50.00 | >50.00 | 49.59 | >50.00 | >50.00 | >50.00 |
| DQ435 | D | 11.73 | 10.11 | 14.47 | >50.00 | >50.00 | >50.00 | >50.00 | >50.00 | >50.00 | >50.00 | >50.00 | >50.00 | >50.00 | >50.00 | >50.00 | >50.00 | 35.91 |
| SF162.LS | B | 0.11 | 0.35 | 0.27 | 0.47 | 0.76 | 0.47 | 0.47 | 0.49 | 0.42 | 0.9 | 0.89 | 0.74 | 0.81 | 0.59 | 0.72 | 1.05 | 0.45 |
| SS1196.1 | B | 0.53 | 0.39 | 0.31 | 2.18 | 1.43 | 1.29 | 0.91 | 0.96 | 1.27 | 1.4 | 1.7 | 1.1 | 1.47 | 0.91 | 1.82 | 1.69 | 0.85 |
| Breadth | N=15 Titer<50 | 53 | 53 | 46 | 27 | 13 | 13 | 13 | 27 | 13 | 13 | 13 | 13 | 13 | 27 | 13 | 13 | 27 |
| Geometric Mean | | 5.181 | 4.706 | 4.072 | 6.607 | 1.042 | 0.779 | 0.654 | 5.243 | 0.730 | 1.122 | 1.230 | 0.902 | 1.091 | 5.302 | 1.145 | 1.332 | 4.407 |

| Virus ID | IA3_Q 105R | IA3_T1 10S | IA3_A 112T | IA3_S 113A | IA1 | CH103 | CH103 VH_T23S | CH103 VH_E56H | CH103 VH_S107I | CH103 VL_26DKV2 8 | CH103 VL_T31V | CH103 VL_E45D | CH103 VL_I48M | CH103 VL_F49Y | CH103 VL_I81T | CH103 VL_D85E | CH103 VH_E56H& VL_E45D | CH106 |
|---|---|---|---|---|---|---|---|---|---|---|---|---|---|---|---|---|---|---|
| 398F1 | >50.00 | >50.00 | >50.00 | >50.00 | >50.00 | >50.00 | 45.56 | >50.00 | 35.56 | >50.00 | >50.00 | 9.23 | 21.65 | 45.39 | 47.52 | 30.01 | >50.00 | >50.00 |
| CNE55 | >50.00 | >50.00 | >50.00 | >50.00 | >50.00 | >50.00 | >50.00 | >50.00 | >50.00 | >50.00 | >50.00 | >50.00 | >50.00 | >50.00 | >50.00 | >50.00 | >50.00 | >50.00 |
| CNE8 | >50.00 | >50.00 | >50.00 | >50.00 | >50.00 | >50.00 | >50.00 | >50.00 | >50.00 | >50.00 | >50.00 | >50.00 | >50.00 | >50.00 | >50.00 | >50.00 | >50.00 | >50.00 |
| TRO11 | >50.00 | >50.00 | >50.00 | >50.00 | 19.95 | 6.35 | 4.86 | 5.58 | 8.71 | 10.92 | 10.27 | 7.63 | 6.42 | 8.07 | 7.87 | 6.61 | 9.06 | 14.05 |
| X2278 | >50.00 | >50.00 | >50.00 | >50.00 | 2 | 4.77 | 4.26 | 0.84 | 4.49 | 20.51 | 18.23 | 3.06 | 4.2 | 5.05 | 4.34 | 3.61 | 1.35 | 0.94 |
| BJOX200 | >50.00 | >50.00 | >50.00 | >50.00 | 47.85 | 24.34 | 18.15 | 19.38 | 18.11 | 23.61 | 26.57 | 18.03 | 21.51 | 24.05 | 25.03 | 21.43 | 32.42 | >50.00 |
| CH119 | >50.00 | >50.00 | >50.00 | >50.00 | >50.00 | >50.00 | >50.00 | >50.00 | >50.00 | >50.00 | >50.00 | >50.00 | >50.00 | >50.00 | >50.00 | 44.84 | >50.00 | >50.00 |
| Ce1176 | >50.00 | >50.00 | >50.00 | >50.00 | >50.00 | >50.00 | >50.00 | >50.00 | >50.00 | >50.00 | >50.00 | >50.00 | >50.00 | >50.00 | >50.00 | >50.00 | >50.00 | >50.00 |
| Ce0217 | >50.00 | >50.00 | >50.00 | >50.00 | 46.64 | 28.95 | 26.65 | 40.64 | 20.19 | 24.77 | 27.27 | 25.15 | 24.93 | 27.93 | 30.05 | 24.58 | >50.00 | >50.00 |
| 25710 | >50.00 | >50.00 | >50.00 | >50.00 | >50.00 | >50.00 | >50.00 | >50.00 | >50.00 | >50.00 | >50.00 | >50.00 | >50.00 | >50.00 | >50.00 | >50.00 | >50.00 | >50.00 |
| X1632 | >50.00 | >50.00 | >50.00 | >50.00 | 20.2 | 3.37 | 2.82 | 18.57 | 4.09 | 9.32 | 8.6 | 1.77 | 3.66 | 4.12 | 4.12 | 3.7 | 14.89 | 24.23 |
| Q842 | >50.00 | >50.00 | >50.00 | >50.00 | 7.39 | 1.85 | 1.62 | 7.07 | 3.35 | 5.55 | 5.35 | 1.55 | 2.58 | 2.98 | 3.18 | 2.81 | 9.44 | 5.79 |
| DQ435 | >50.00 | >50.00 | >50.00 | >50.00 | 9.52 | 14.01 | 11.2 | 5.86 | 12.35 | 14.82 | 16.12 | 12.45 | 11.33 | 11.39 | 12.93 | 11.6 | 2.44 | 6.31 |
| SF162.LS | 0.84 | 0.49 | 0.5 | 0.73 | 0.18 | 0.29 | 0.29 | 0.11 | 0.27 | 0.5 | 0.67 | 0.34 | 0.29 | 0.25 | 0.31 | 0.33 | 0.08 | 0.08 |
| SS1196.1 | 1.1 | 1.33 | 1.62 | 1.43 | 0.39 | 1.05 | 0.73 | 0.26 | 1.05 | 1.31 | 1.18 | 0.6 | 1.25 | 1.16 | 1.32 | 1.16 | 0.51 | 0.24 |
| Breadth | 13 | 13 | 13 | 13 | 60 | 60 | 67 | 60 | 67 | 60 | 60 | 67 | 67 | 67 | 67 | 73 | 53 | 47 |
| Geometric Mean | 0.961 | 0.807 | 0.900 | 1.022 | 5.916 | 4.359 | 4.671 | 3.512 | 5.479 | 7.390 | 7.548 | 3.831 | 5.110 | 5.892 | 6.205 | 6.433 | 2.938 | 2.167 |

Geometric means were calculated for neutralization sensitive viruses with an IC50 value <50μg/ml.

| 0.01-1.0 | 1.0-10.0 | 10.0-50.0 | >50 |
|---|---|---|---|

**Table S3. Summary of poly-/autoreactivity of the CH103-106 lineage antibody mutants and controls.**

| Antibody | | Assays | | | |
|---|---|---|---|---|---|
| | | ELISA | HEp-2 cell staining | Protein microarray | UBE3A binding |
| IA2 mutant abs | IA2_N60S | | ± | | |
| | IA2_E64K | + | ++ | + | ++ |
| | | | | | |
| IA3 mutant abs | IA3_L11V | | | | |
| | IA3_Y32T | | | | |
| | IA3_Q39L | | | | |
| | IA3_V42G | | | | |
| | IA3_N60S | | | | |
| | IA3_E64K | | | | ++ |
| | IA3_S65G | | | | |
| | IA3_T68S | | | | |
| | IA3_V69I | | | | |
| | IA3_N76D | | | | |
| | IA3_Y91F | | | | |
| | IA3_F99Y | | | | |
| | IA3_D101R | | | | |
| | IA3_Q105R | | | | |
| | IA3_T110S | | | | |
| | IA3_A112T | | | | |
| | IA3_S113A | | | | |
| | | | | | |
| CH103 mutant abs | CH103 VH_T23S | | | | + |
| | CH103 VH_E56H | + | ± | | ++ |
| | CH103 VH_S107I | | | | + |
| | CH103 VL_26DKV28 | | | | + |
| | CH103 VL_T31V | | | | + |
| | CH103 VL_E45D | | | | ++ |
| | CH103 VL_I48M | | | | + |
| | CH103 VL_F49Y | | | | + |
| | CH103 VL_I81T | | | | + |
| | CH103 VL_D85E | | | | + |
| | | | | | |
| Parental abs | IA1 | + | +++ | + | + |
| | IA2 | | | | |
| | IA3 | | | | |
| | CH103 | | | | + |
| | CH106 | + | ++ | | ++ |
